# Supplementary material for: Predicting visual function by interpreting a neuronal wiring diagram
Source: Nature. 2024 Oct 2;634(8032):113–23. doi: 10.1038/s41586-024-07953-5 (PMC11446822; doi:10.1038/s41586-024-07953-5)
Supplement: Supplementary file 6 — CRF and ERF predictions for individual TmY4 and TmY9 cells. Analogous to Supplementary Data 3, but for TmY target types. Shown are the top four monosynaptic pathways, the strongest pathway passing through each of the top ten intermediary types (ranking from Extended Data Fig. 7), and the trisynaptic pathway Tm1–TmY–Dm3–TmY (see the section entitled Prediction of spatial normalization). [file 41586_2024_7953_MOESM6_ESM.zip › DataS4/TmY9qΓèÑ/720575940627190149.pdf]

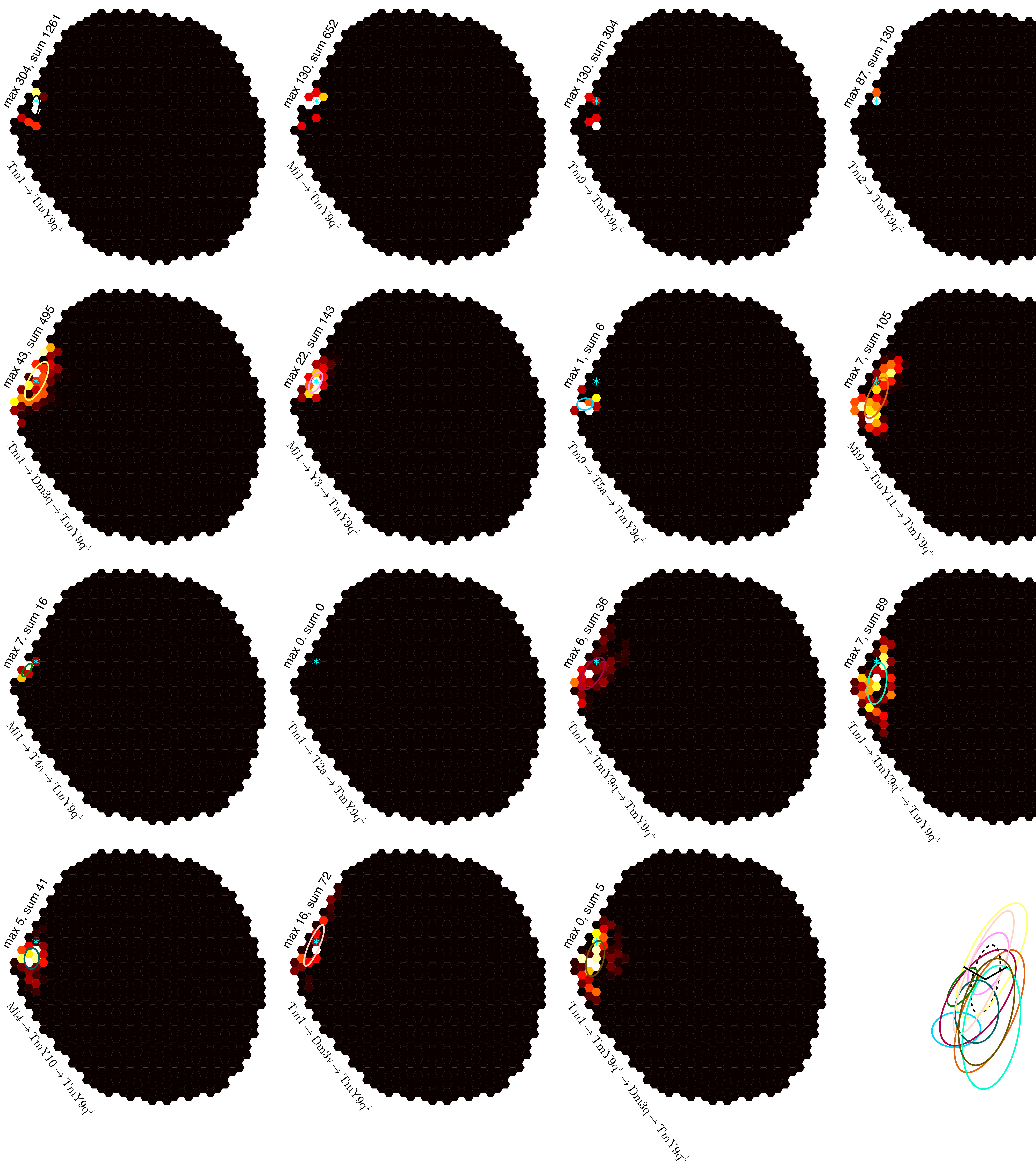

max 304, sum 1261
Tm1 → TmY9q⊥
max 130, sum 652
Mi1 → TmY9q⊥
max 130, sum 304
Tm9 → TmY9q⊥
max 87, sum 130
Tm2 → TmY9q⊥
max 43, sum 495
Tm1 → Dm3q → TmY9q⊥
max 22, sum 143
Mi1 → Y3 → TmY9q⊥
max 1, sum 6
Tm9 → T5a → TmY9q⊥
max 7, sum 105
Mi9 → TmY11 → TmY9q⊥
max 7, sum 16
Mi1 → T4a → TmY9q⊥
max 0, sum 0
Tm1 → T2a → TmY9q⊥
max 6, sum 36
Tm1 → TmY9q → TmY9q⊥
max 7, sum 89
Tm1 → TmY9q⊥ → TmY9q⊥
max 5, sum 41
Mi4 → TmY10 → TmY9q⊥
max 16, sum 72
Tm1 → Dm3v → TmY9q⊥
max 0, sum 5
Tm1 → TmY9q⊥ → Dm3q → TmY9q⊥
